# Preclinical activity of selinexor, an inhibitor of XPO1, in sarcoma

**DOI:** 10.18632/oncotarget.7667

**Published:** 2016-02-24

**Authors:** Robert Nakayama, Yi-Xiang Zhang, Jeffrey T. Czaplinski, Alex J. Anatone, Ewa T. Sicinska, Jonathan A. Fletcher, George D. Demetri, Andrew J. Wagner

**Affiliations:** ^1^ Ludwig Center at Dana-Farber/Harvard and Center for Sarcoma and Bone Oncology, Department of Medical Oncology, Harvard Medical School, Boston, MA, USA; ^2^ Department of Orthopaedic Surgery, School of Medicine, Keio University, Tokyo, Japan; ^3^ Department of Medical Oncology and Center for Molecular Oncologic Pathology, Dana-Farber Cancer Institute, Harvard Medical School, Boston, MA, USA; ^4^ Department of Pathology, Brigham and Women's Hospital, Harvard Medical School, Boston, MA, USA

**Keywords:** sarcoma, gastrointestinal stromal tumor, liposarcoma, selinexor, preclinical study

## Abstract

Selinexor is an orally bioavailable selective inhibitor of nuclear export that has been demonstrated to have preclinical activity in various cancer types and that is currently in Phase I and II clinical trials for advanced cancers. In this study, we evaluated the effects of selinexor in several preclinical models of various sarcoma subtypes. The efficacy of selinexor was investigated *in vitro* and *in vivo* using 17 cell lines and 9 sarcoma xenograft models including gastrointestinal stromal tumor (GIST), liposarcoma (LPS), leiomyosarcoma, rhabdomyosarcoma, undifferentiated sarcomas, and alveolar soft part sarcoma (ASPS). Most sarcoma cell lines were sensitive to selinexor with IC_50_s ranging from 28.8 nM to 218.2 nM (median: 66.1 nM). Selinexor suppressed sarcoma tumor xenograft growth, including models of ASPS that were resistant *in vitro*. In GIST cells with KIT mutations, selinexor induced G_1_- arrest without attenuation of phosphorylation of KIT, AKT, or MAPK, in contrast to imatinib. In LPS cell lines with *MDM2* and *CDK4* amplification, selinexor induced G_1_-arrest and apoptosis irrespective of p53 expression or mutation and irrespective of RB expression. Selinexor increased p53 and p21 expression at the protein but not RNA level, indicating a post-transcriptional effect. These results indicate that selinexor has potent *in vitro* and *in vivo* activity against a wide variety of sarcoma models by inducing G_1_-arrest independent of known molecular mechanisms in GIST and LPS. These studies further justify the exploration of selinexor in clinical trials targeting various sarcoma subtypes.

## INTRODUCTION

XPO1 is a member of the Karyopherin β superfamily of nuclear transport proteins that facilitates the nuclear export of RNA [1] and cargo proteins with leucine-rich nuclear export signals (NESs) by forming a ternary complex with Ran-GTP [2]. These NES-bearing cargo proteins include tumor suppressors such as p53 [3, 4], RB [5], and APC [6], cell cycle regulators such as p21 [7, 8], and many others [9–11]. While it prominently accumulates at the nuclear envelope in interphase, XPO1 localizes to kinetochores and also plays a role in mitotic progression and chromosome segregation together with Ran-GTP, as the nuclear envelope breaks down in prometaphase during mitosis [12].

XPO1 overexpression has been associated with chemo-resistance and poor prognosis of several cancers [13–16]. Although their role in tumor development or progression remains to be elucidated, recurrent mutations in XPO1 have been identified in chronic lymphoblastic leukemia [17, 18]. The classic XPO1 inhibitor Leptomycin B [19, 20] is cytotoxic *in vitro* and *in vivo* [21], and disrupts mitotic progression and chromosome segregation [12]. Selective inhibitors of nuclear export (SINEs) have been designed to bind covalently to human XPO1 at Cys528 in the NES-binding groove, thereby irreversibly inhibiting the binding to target proteins and a subsequent ternary complex formation [22, 23]. Selinexor (KPT330) is an orally bioavailable SINE currently in clinical development. Prior preclinical and clinical studies have demonstrated activity in certain solid tumors [24–28] as well as in hematologic malignancies [29–31] with induction of cell cycle arrest or apoptosis and nuclear accumulation of XPO1 cargo tumor suppressor proteins.

Sarcomas constitute a heterogeneous group of malignant mesenchymal tumors. Effective small molecule targeted therapies have been established only in a small subset of this group with defined molecular backgrounds, such as imatinib for mutated KIT in gastrointestinal stromal tumors (GIST) [32, 33]. Cytotoxic agents remain first line chemotherapy for the vast majority of high grade sarcomas and the discovery of novel therapeutic approaches is needed. In this study, we evaluated the efficacy of selinexor in several preclinical models of various sarcoma subtypes.

## RESULTS

### Cell viability assays

**Figure 1:**
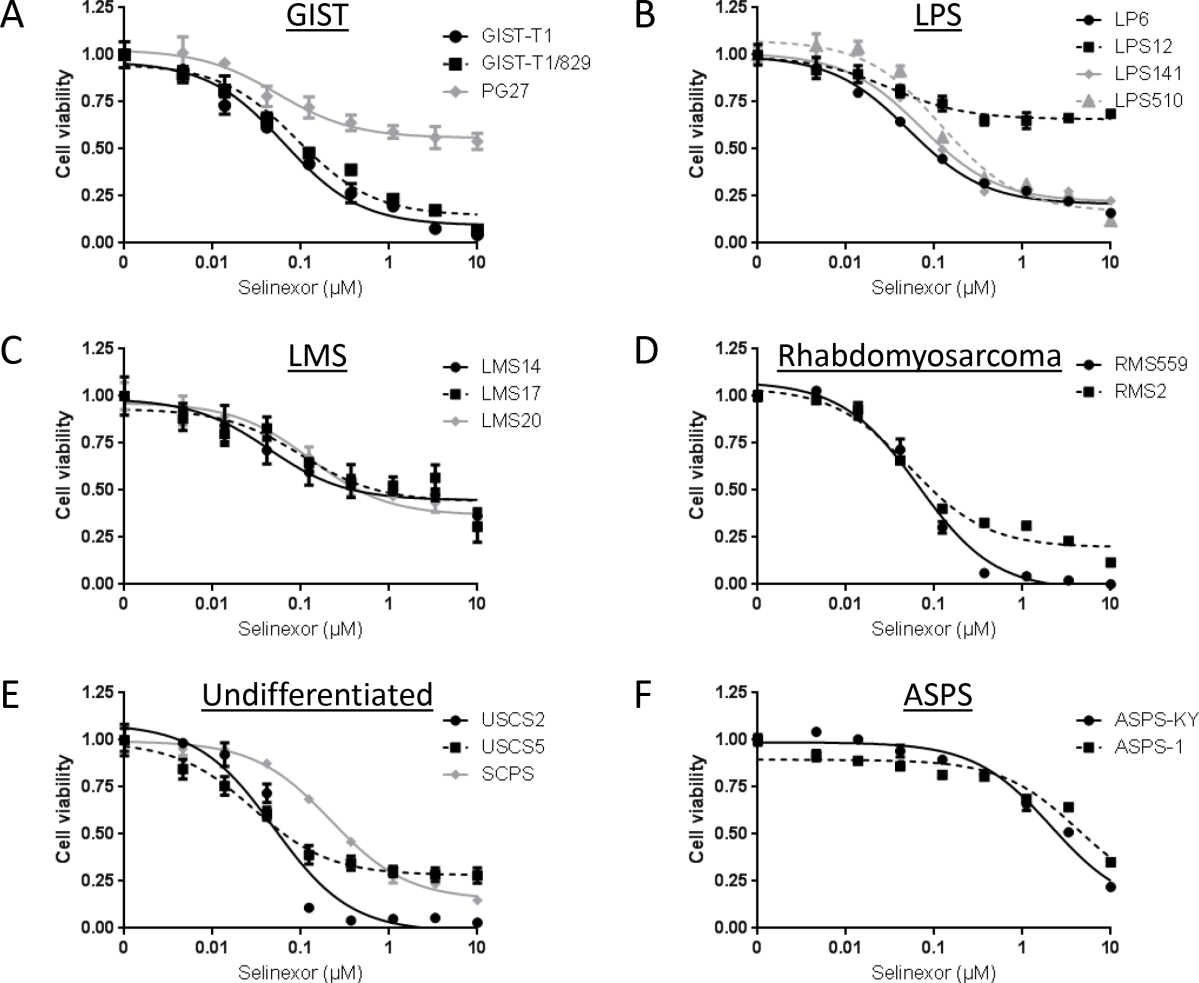
Anti-proliferative activity of selinexor in a variety of sarcoma cell lines *in vitro* Cell viability was measured using Cell Titer Glo Luminescent Cell Viability Assay Kit. (**A**) GIST cell lines. (**B**) LPS cell lines. (**C**) LMS cell lines. (**D**) Rhabdomyosarcoma cell lines. (**E**) Undifferentiated sarcoma cell lines. (**F**) ASPS cell lines.

We first conducted *in vitro* cell viability assays using Cell Titer Glo following 72-hour treatment of a wide variety of sarcoma cell lines with selinexor (Figure 1, Supplementary Table 1). Most cell lines were sensitive to selinexor with IC_50_s ranging from 28.8 nM to 218.2 nM (median: 66.1 nM). Among these, the ASPS lines, ASPS-KY and ASPS-1, were exceptionally resistant to selinexor with IC_50_ greater than 2 μM. Some cell lines, such as LPS12, showed shallow curves; this is likely due to their slow growth rates since the cell viability curves shifted deeper with almost identical relative IC_50_s when treated for seven days (data not shown). These data demonstrate that many but not all sarcoma histologic subtypes are sensitive to selinexor *in vitro*.

### Selinexor suppresses tumor growth in human sarcoma xenograft models

**Figure 2:**
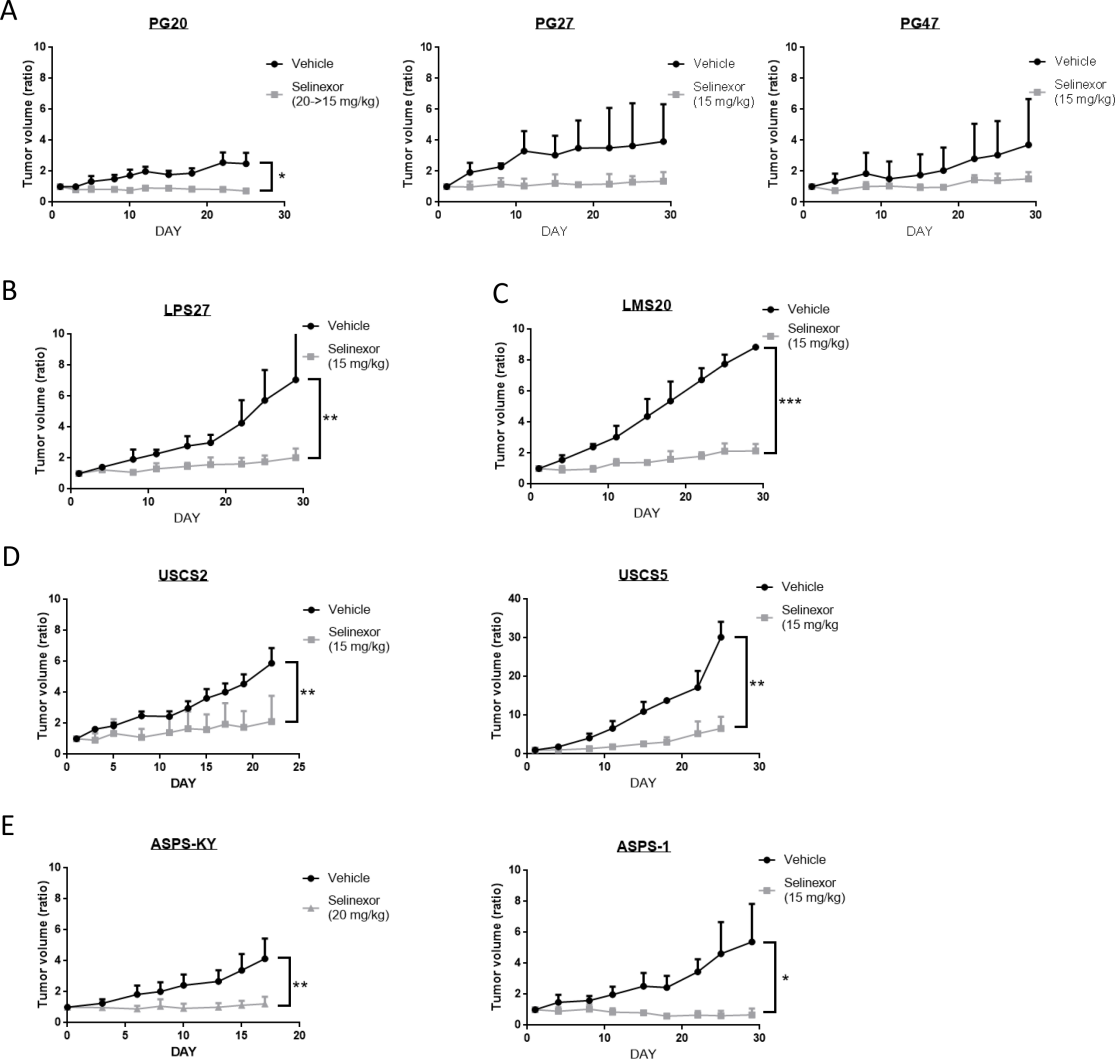
Antitumor activity of selinexor in a variety of sarcoma models *in vivo* Three mice were treated either with control or selinexor in each cohort. The size of subcutaneously implanted tumors was evaluated by measuring the long and short diameters. The Y-axis indicates average changes in volume from day 1. (**A**) GIST models. (**B**) LPS models. (**C**) LMS models. (**D**) Undifferentiated sarcoma models. (**E**) ASPS models.

To investigate if selinexor exhibits antitumor activity in a more physiologically relevant setting, we used 9 human sarcoma xenograft models. We finally determined to treat the mice at 15 mg/kg twice weekly, as treatment at 20 mg/kg twice weekly led to severe weight loss (> 10% of their weight) in 3 out of 7 mice (PG20 and ASPS-KY), while severe weight loss was not observed when treated at 15 mg/kg (Supplementary Figure 1). Selinexor administered at 15 mg/kg twice weekly significantly suppressed tumor growth compared to tumors in mice treated with vehicle alone. Although ASPS models were exceptionally resistant to selinexor *in vitro* (Figure 1F), xenograft models showed *in vivo* sensitivity comparable to other sarcoma models (Figure 2E). These data demonstrate that selinexor has *in vivo* activity in all models tested.

### Histological findings

**Figure 3:**
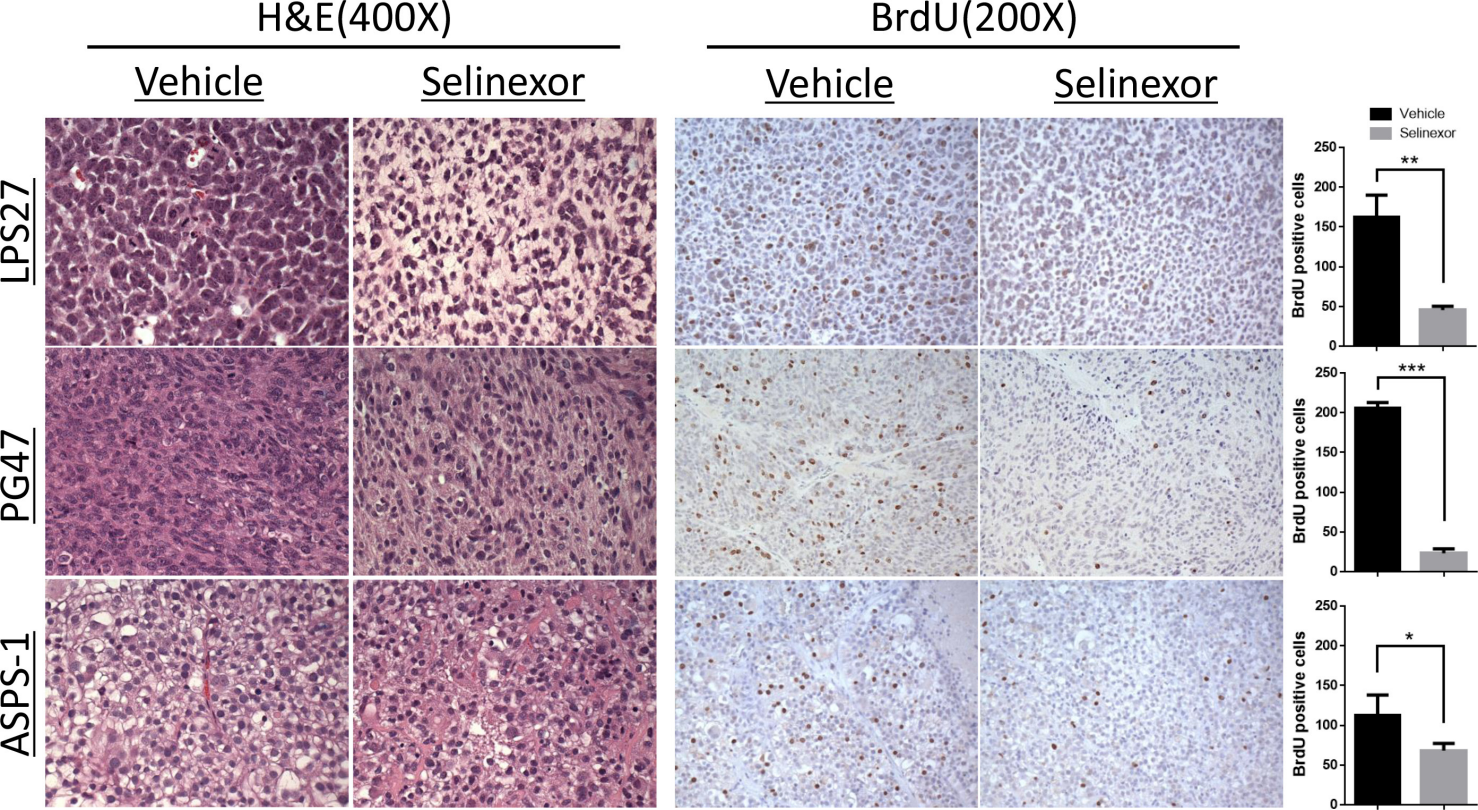
Histological changes and reduced cell proliferation following selinexor treatment BrdU solution was injected intraperitoneally 22 hours after the last drug administration. After 2 additional hours, tumors were harvested and fixed for histologic analysis. BrdU positive cells were counted in three representative fields at 200× magnification and compared between two groups (right bar graphs). LPS27, the tumor cells treated with selinexor showed smaller nuclei, some with a pyknotic appearance, and abundant clear cytoplasm, whereas the control tumors showed sheets of large round cells with vesicular chromatin, prominent nucleoli, frequent mitotic figures, and minimal cytoplasm; PG47 (GIST), the treated tumor did not show any appreciable difference in H & E; ASPS-1, the treated tumor showed loss of delicate capillary vasculature and alveolar/nested architecture and there were areas of smaller cells with a more compact appearance.

Tumors were harvested and fixed for subsequent histologic analysis following treatment of either vehicle or selinexor (Figure 3). LPS27 showed a dramatic change in histologic appearance following treatment. Control tumors showed sheets of large round cells with vesicular chromatin and minimal cytoplasm with frequent mitotic figures whereas tumors treated with selinexor showed smaller nuclei and abundant clear cytoplasm. In most models, selinexor-treated tumors tended to be less cellular but demonstrated little appreciable difference in morphology when compared with control tumors, like PG47 (GIST) representatively shown in Figure 3. In ASPS models, the treated tumors showed a loss of delicate capillary vasculature and alveolar/nested architecture and there were areas of smaller cells with a more compact appearance. Cell proliferation as measured by BrdU incorporation was significantly suppressed in all the models tested (Figure 3). Assessment of apoptosis by TUNEL assay in LPS and ASPS models did not show any significant difference between two groups (data not shown).

### Effects on KIT, p53, and RB signaling pathways

Cell lines from several sarcoma subtypes with defined molecular backgrounds, such as GIST with *KIT* mutations and dedifferentiated LPS with *MDM2* and *CDK4* amplification, were treated with selinexor to investigate potential mechanisms of action.

### Selinexor induces cell cycle arrest in GIST independent of alterations in the *KIT* signaling pathway

**Figure 4:**
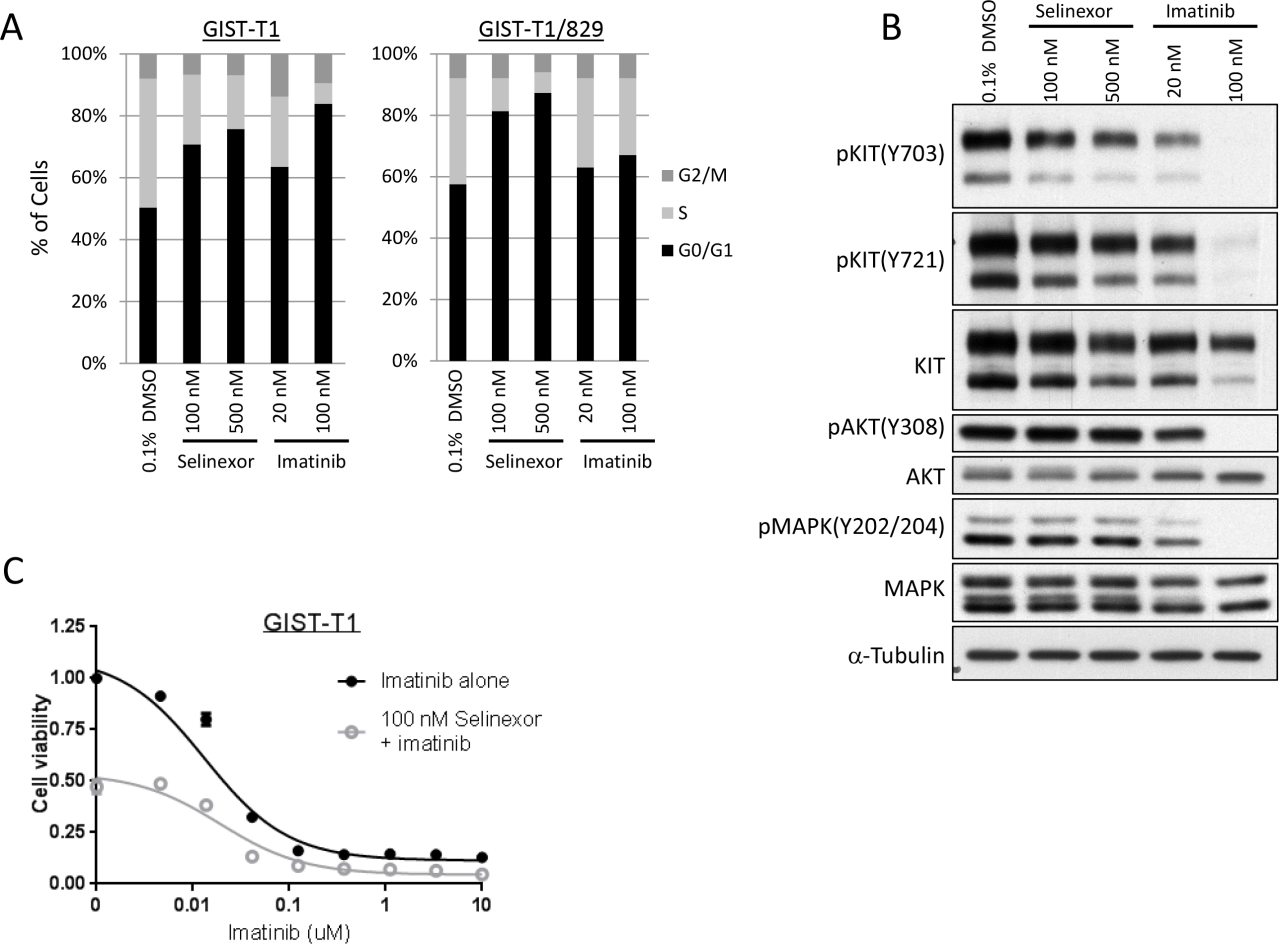
Selinexor induced cell cycle arrest in GIST independent of KIT signaling pathway (**A**) Cell cycle analysis by propidium iodide staining in the GIST-T1 line and the GIST-T1/829 subclone. The cells were fixed following 24-hour exposure of each drug and analyzed by flow cytometry. (**B**) Protein expression analysis in the GIST-T1 line following 24-hour exposure of each drug. (**C**) Cell viability assay in the GIST-T1 line following the 72-hour exposure to the serial concentration of imatinib (IM) with or without 100 nM selinexor.

The majority of GIST is driven by mutations in the receptor tyrosine kinase *KIT* and corresponding constitutive activation of signaling pathways [34]. We investigated the mechanism of action of selinexor with particular attention to the phosphorylation status of KIT and its downstream pathways using a KIT-mutant cell line, GIST-T1, and its imatinib-resistant subclone, GIST-T1/829, which contains a secondary mutation in *KIT* [35]. In cell viability assays, selinexor showed similar activity against GIST-T1 and GIST-T1/829 (Supplementary Table 1 and Figure 1A). The cells were exposed to 100 nM and 500 nM of selinexor in the subsequent experiments, roughly equivalent to the IC_50_ and IC_75_, respectively. In cell cycle analyses, selinexor induced G_1_-arrest in a dose-dependent manner irrespective of the presence of secondary *KIT* mutation, while imatinib induced G_1_-arrest only in the naive GIST-T1 line and showed little activity against GIST-T1/829 (Figure 4A). Western blotting showed that selinexor slightly decreased the total protein expression of KIT and phosphorylated KIT but exhibited no effect on the phosphorylation of downstream molecules (AKT and MAPK) in GIST-T1 cells, whereas imatinib caused a dramatic decrease in phosphorylation of KIT as well as of downstream molecules (Figure 4B). The combination of selinexor and imatinib showed an additive effect in cell viability assays (Figure 4C). The above data suggested that these drugs work through different, parallel pathways.

### Selinexor induces cell cycle arrest and apoptosis in dedifferentiated LPS differently from Nutlin-3a and independently of p53 and RB

**Figure 5:**
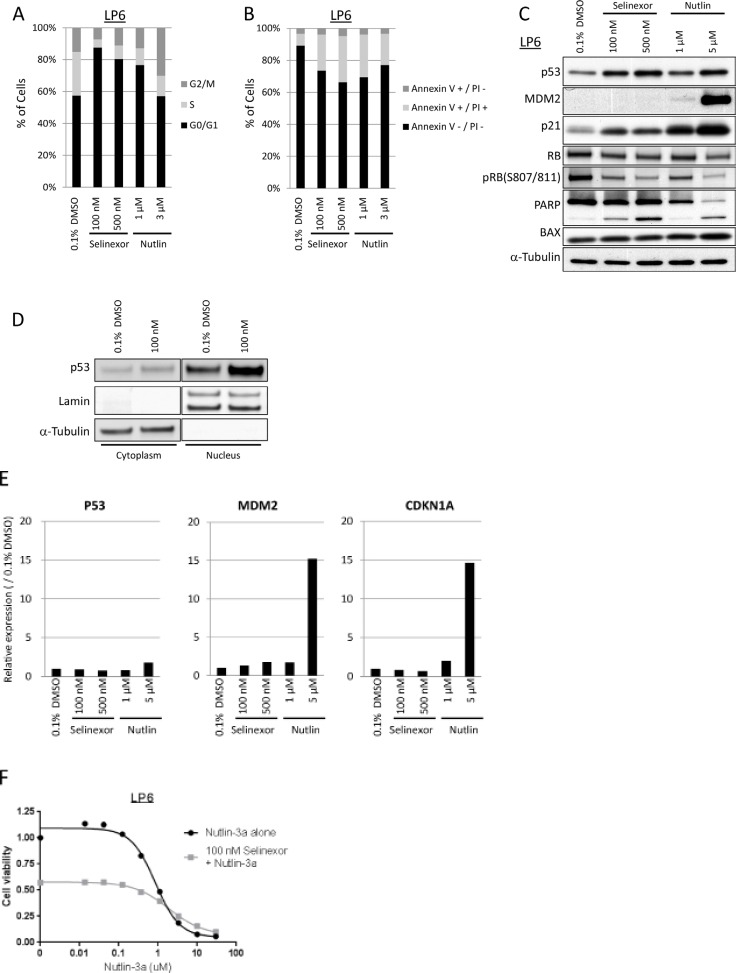
Selinexor induced cell cycle arrest and apoptosis in LPS differently from Nutlin-3a (**A**) Cell cycle analysis by propidium iodide staining in the LP6 line. The cells were fixed following 24-hour exposure of each drug. (**B**) Apoptosis analysis by annexin V/propidium iodide staining in the LP6 line. The cells were stained following 24-hour exposure of each drug. (**C**) Protein expression analysis in the LP6 line following 24-hour exposure of each drug. (**D**) Nuclear localization of p53 following 24-hour exposure with 100 nM Selinexor. (**E**) Gene expression analysis of p53, MDM2 and CDKN1A (gene encoding p21) in the LP6 by qPCR. Total RNA was extracted following 24-hour exposure of each drug. Expression at the transcription in each condition was normalized to the one treated with 0.1% DMSO. (**F**) Cell viability assay in the LP6 line following the 72-hour exposure to the serial concentration of Nutlin-3a with or without 100 nM selinexor.

Since both p53 and p21 bear NESs and are exported out of the nucleus by XPO1, we hypothesized that selinexor might enhance their activities by maintaining nuclear localization. To address this hypothesis, we tested the *in vitro* effects of selinexor in a dedifferentiated LPS cell line, LP6, which harbors high copy number of *MDM2*, and compared it to the effects of a classic MDM2 inhibitor, Nutlin-3a [36], which was used as a positive control. Selinexor increased the G_1_ population in cell cycle analysis (Figure 5A), and increased the Annexin V-positive population (Figure 5B), indicating that it induced both G_1_-arrest and apoptosis in LP6 cells at 100 nM, equivalent to the IC_50_ in LP6 in the cell viability assay (Table 1 and Figure 1). Western blotting showed an increase in p53 and p21 protein expression, but no significant change in expression of MDM2, in contrast to the effects of Nutlin-3a which also induced MDM2 (Figure 5C) following selinexor treatment. p53 expression increased more significantly in the nucleus than in cytoplasm (Figure 5D). Phosphorylation of RB decreased following exposure to selinexor as well as to Nutlin-3a, coincident with induction of the cyclin-dependent kinase inhibitor p21 (Figure 5C). PARP cleavage, an indicator of apoptosis, was increased in a dose-dependent manner, although significant changes in the p53 transcriptional target BAX expression were not observed (Figure 5C).

Quantitative RT-PCR showed no increase in p53, MDM2, or CDKN1A RNA level following treatment of LP6 with selinexor (Figure 5E) whereas significant induction of MDM2 and CDKN1A RNA expression followed treatment with Nutlin-3a. The combination of Nutlin-3a and selinexor did not show a significant additive effect in the cell viability assay (Figure 5F), suggesting that the effectors of selinexor treatment may overlap with those of Nutlin-3a in MDM2-amplified LPS.

**Figure 6:**
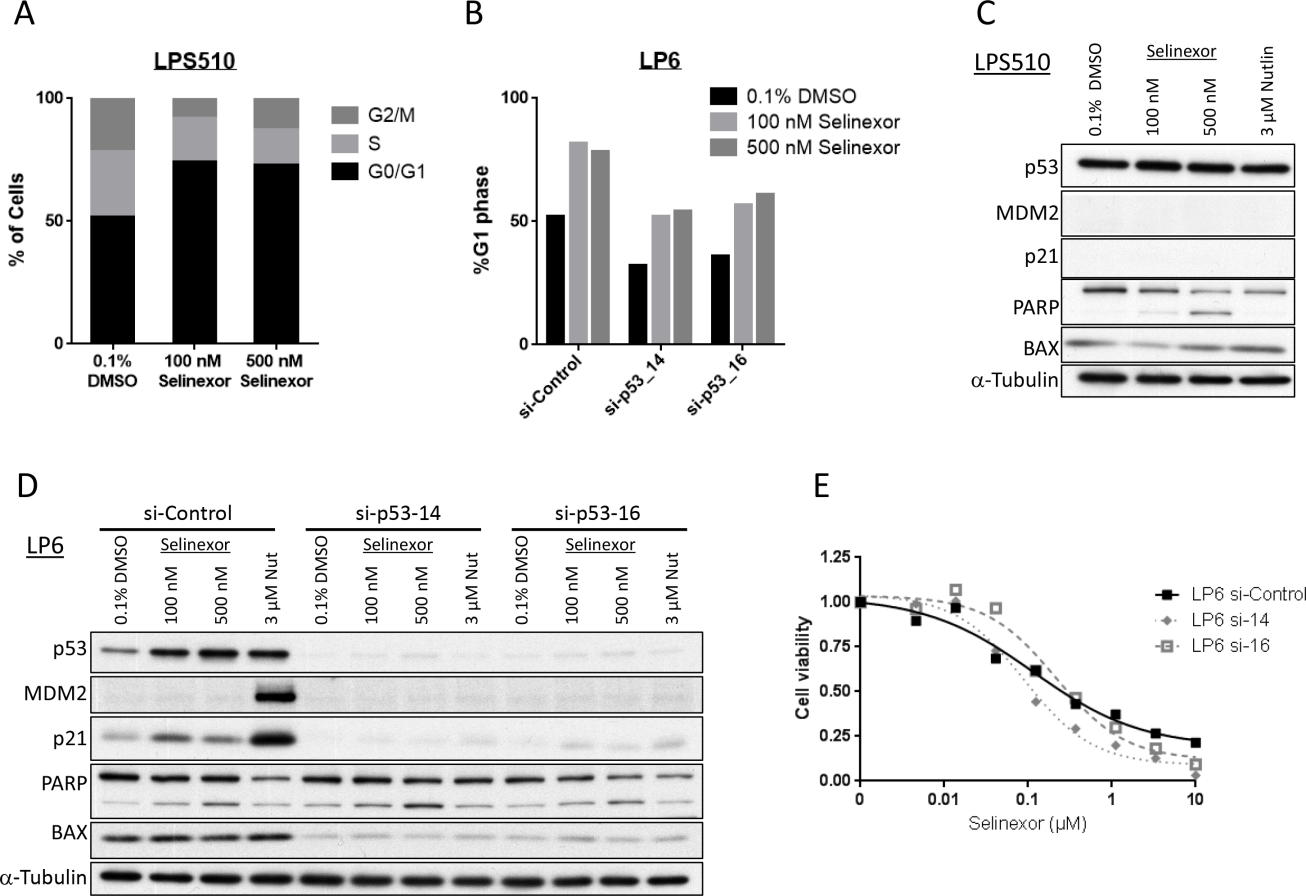
Selinexor acts independently of p53 in LPS (LPS510 and p53 knocked-down LP6) (**A**) Cell cycle analysis by propidium iodide staining in the p53-mutant LPS510 line. The cells were fixed following 24-hour exposure of each drug. (**B**) Cell cycle analysis by propidium iodide staining in the LP6 lines that were treated with control or p53 siRNA. The cells were fixed following 24-hour exposure of each drug. (**C**) Protein expression analysis in the p53-mutant LPS510 line following 24-hour exposure of each drug. (**D**) Protein expression analysis in the LP6 lines transfected with control or p53 siRNA following 24-hour exposure of each drug. (**E**) Cell viability assay in the p53-knocked down LP6 lines following the 72-hour exposure to the serial concentration of selinexor.

To address if G_1_-arrest and apoptosis induced by selinexor in LPS are dependent on the status of p53, we analyzed the activity of selinexor in two other models: a p53 mutant LPS line, LPS510 and the LP6 line with p53-knocked down by siRNA treatment. Cell cycle analysis again showed a significant increase in G_1_ phase following treatment with selinexor in both models (Figure 6A, 6B). Western blotting showed PARP cleavage induced by selinexor in a dose-dependent manner in both models, without significant increase in p53 protein expression or significant expression of p21 and MDM2 (Figure 6C, 6D). Knockdown of p53 led to minimal changes in the shape of the cell viability curve and IC_50_ following treatment with selinexor (Figure 6E), whereas it caused a significant change following treatment with Nutlin-3a (Supplementary Figure 2). These data demonstrate that selinexor induces cell cycle arrest and apoptosis through p53-indpendent mechanisms in LPS models. Similarly, siRNA-mediated knockdown of RB in LP6 did not result in any significant change in cell cycle arrest induced by selinexor (Supplementary Figure 3), suggesting that its mechanism of action is also independent of RB, in contrast with the RB-dependent effects of CDK inhibitors [37].

## DISCUSSION

In this study, we demonstrated the *in vitro* and *in vivo* antitumor activity of selinexor, a selective inhibitor of XPO1, in sarcomas using 17 cell lines and 9 xenograft models. A number of preclinical studies of selinexor and other SINEs in different tumor models have been reported so far. Selinexor has been demonstrated to inhibit tumor growth *in vitro* with IC_50_ ranging from 10 nM to 1 μM both in hematologic malignancies [30] and solid tumors [24, 25, 38], with wide therapeutic windows [39, 40]. The results from the current study, where IC_50_s ranged from 28.8 nM to 218.2 nM (median: 66.1 nM), were comparable to those in previous studies. In addition, selinexor demonstrated significant antitumor activity in all xenograft models in this study.

In general, in the experiments presented here, selinexor appeared to exhibit a growth arrest rather than cytotoxic activity as indicated by the curves in Figure 2 and histological findings in Figure 3. Of note, the difference in sensitivity of *in vitro* and *in vivo* ASPS models suggests a potential effect on the tumor-stromal interaction or anchorage-independent growth of the tumor.

A number of possible mechanisms of action of SINEs have been suggested, since XPO1 is known to be engaged in nuclear export of numerous cargo proteins, including the involvement of many tumor suppressor gene products [11, 24, 38, 41]. Sarcomas are highly heterogeneous both histologically and genetically. Thus, we considered that it would be difficult to identify a common mechanism of selinexor in sarcoma models and focused on investigating this in more detail in cell lines from two subtypes with defined molecular backgrounds, GIST and dedifferentiated LPS. The results in the study indicated that although we could not identify the specific mechanism of action in GIST, selinexor worked through mechanisms completely independent of the KIT signaling pathway and therefore use of selinexor may represent a novel approach to the treatment of KIT inhibitor-resistant disease.

The majority of well differentiated and dedifferentiated LPS harbor genomic amplification of 12q13-15 resulting in overexpression of the genes in this region [42, 43]. Among these, overexpression of MDM2, a transcriptional repressor and E3 ubiquitin ligase responsible for the ubiquitination and degradation of p53, has been implicated in tumorigenesis in LPS, and several MDM2 inhibitors are under clinical development [44]. MDM2, p53, and the p53 transcriptional target p21 all harbor a nuclear export signal in their structure, and they are exported by XPO1 from the nucleus to the cytoplasm [45, 46]. We hypothesized that selinexor could inhibit nuclear to cytoplasmic shuttling of MDM2, and subsequently stabilize p53, and investigated the activity of selinexor *in vitro* using the LPS lines with MDM2 amplification (LP6, LPS141 and LPS12) and with mutant p53 (LPS510). Selinexor induced both G_1_-arrest and apoptosis in LP6, and protein and gene expression analysis indicated that the increase in p53 and p21 protein was attributed to post-transcriptional modification rather than changes in RNA expression. Despite stabilization of p53 protein there was no induction of transcriptional targets of p53 following exposure to selinexor in these model system. The results in a similar experiment using the p53-mutant LPS510 line and p53-knockdown in LP6 indicated that selinexor was capable of inducing both G_1_-arrest and apoptosis irrespective of the mutation status or expression of p53 in LPS lines. We also observed that selinexor caused G_1_-arrest in LP6 irrespective of RB expression in a similar way.

The results from the *in vitro* experiments suggest that selinexor exhibited its anti-tumor activity independently of the defined pathways in both GIST and LPS. The precise mechanisms of action of selinexor remained to be elucidated but these data raise the possibility that selinexor may be active even in the context of resistance to other targeted agents.

On account of the great diversity of the cargo proteins of XPO1, we hypothesize that SINEs may activate multiple checkpoints that can overcome genetic alterations that create the molecular background of many different types of tumors. Interestingly, we found a difference in activity on combination use of selinexor *in vitro*. The combination of imatinib and selinexor demonstrated an additive effect in GIST, whereas no significant enhancement of the effects of Nutlin-3a and selinexor was observed in LPS. On the contrary, Kojima et al. reported that another SINE, KPT-185, synergized with the MDM2 inhibitor Nutlin-3a to induce p53 and apoptosis in AML [41]. We speculate that selinexor's unique stabilization of p53 protein without induction of p53 transcriptional targets may partially antagonize the effects of Nutlin-3a in the context of MDM2 amplification. Extrapolating from these results, it is possible that selinexor provides an additive effect when used with drugs that work outside the nucleus, such as receptor tyrosine kinase inhibitors.

One limitation of this study is the relatively small cohort sizes for the xenograft studies. By using the minimal number of mice for statistical analyses (*n* = 3) in each experimental group, we were able to test the activity of selinexor in a wide variety of xenograft models (*n* = 9). Using more mice per cohort may have provided further statistical confidence in our observations but nonetheless the differences compared to the control group were evident with this cohort size. The small cohort size limited our ability to observe effects of prolonged treatment or of regrowth after treatment cessation, since all tumors were used at the end of the treatment to focus on immunohistochemical and BrdU anslyses.

In conclusion, selinexor has potent *in vitro* and *in vivo* activity against a wide variety of sarcoma models. Selinexor induced G_1_-arrest independent of known molecular mechanisms in GIST and LPS. These studies further justify the exploration of selinexor in clinical trials targeting various sarcoma subtypes.

## MATERIALS AND METHODS

### Cell lines

The efficacy of selinexor was investigated *in vitro* using 17 cell lines including GIST, liposarcoma (LPS), leiomyosarcoma (LMS), rhabdomyosarcoma, alveolar soft part sarcoma (ASPS), and undifferentiated sarcomas (Supplementary Table 1) [47–49]. Of these, 13 cell lines and an imatinib-resistant sub-clone of GIST-T1, GIST-T1/829, were established at Brigham and Women's Hospital or Dana-Farber Cancer Institute [35]. GIST-T1 (39) was generously provided by Dr. Takahiro Taguchi. ASPS-KY (40) was obtained from an ASPS support group in 2009 with the permission of Dr. Shunsuke Yanoma. ASPS-1 (41) was purchased from DCTD Tumor/cell line Repository at the NCI at Frederick in 2013. All cell lines have been characterized by high-resolution short tandem repeat profiling with Promega PowerPlex 1.2 system at the Molecular Diagnostics Laboratory of Dana-Farber Cancer Institute. The cells used for the experiment are passaged for less than 6 months after authentication. Cell lines were cultured in DMEM/F12 medium (Gibco, Grand Island, NY, USA), supplemented with 10% fetal bovine serum, Glutamax (Gibco) and Antibiotic-Antimycotic (Gibco).

### Xenograft models

The efficacy of selinexor was investigated *in vivo* using 9 sarcoma xenograft models including GIST, LPS, LMS, ASPS, and undifferentiated sarcomas. Tumors used for xenograft studies were obtained from patients undergoing standard care of surgery who consented to research use of material according to an Institutional Review Board-approved protocol. Either cryopreserved tumors or cell lines mixed 1:1 with Matrigel were subcutaneously implanted into the flanks of female nude mice (Nu/Nu; Charles River Laboratories). Tumor volume (V) was estimated using the following equation: V = A × B^2^ × 0.5 (A, long diameter; B, short diameter). Since some of the xenograft tumors grow extremely fast, we began treatment at a relatively early stage when tumors reached an average size of 50−100 mm^3^ in order to be able to observe the long-term effects of selinexor in comparison to vehicle control for up to four weeks. Mice were randomized into statistically identical cohorts (3 mice/group). Selinexor was prepared once a week in 0.6% w/v Pluronic F-68 and 0.6% w/v PVP K-29/32 diluent) and administered twice weekly at the designated dose (14–20 mg/kg) by oral gavage. Tumor size and mouse weight were recorded every 2 to 3 days. Bromodeoxyuridine (BrdU) solution (10 mg/mL, 0.2 mL/mouse) was injected intraperitoneally 22 hours after the last drug administration. After 2 additional hours, mice were sacrificed and tumors were fixed in 10% formalin for immunohistochemistry analysis. BrdU positive cells were counted in three representative fields at 200× magnification. All procedures were carried out according to protocols approved by the Institutional Animal Care and Use Committee of the Dana-Farber Cancer Institute.

### Cell viability assay

Cells were plated at a density of 1,000 to 2,000 cells per well in 100 μl of medium in a 96-well plate. After 24 hours, cells were exposed to 0.1% DMSO or serial dilutions of selinexor (up to 10 mM) for 3 days. Cell viability was measured using Cell Titer Glo Luminescent Cell Viability Assay Kit (Promega, Madison, WI, USA) with a modification in the protocol in that the Cell Titer Glo reagent was diluted 1:3 with PBS. The relative luminescence units (RLU) were measured using FLUOstar Optima plate reader (BMG Labtech GmbH) and relative cell number was calculated by normalization to the RLU of the 0.1% DMSO treated cells.

### Cell cycle analysis

Cells were exposed to selinexor or 0.1% DMSO for 24 hours and harvested. After washing with cold PBS, cells were fixed with 70% ethanol and cryopreserved at −20°C. Fixed cells were stained in PBS containing 10 μg/mL RNase A and 20 μg/mL propidium iodide (Sigma) for 15 min at room temperature in the dark. Cells were analyzed by flow cytometry using BD LSR Fortessa (BD Biosciences, San Jose, CA, USA). The DNA histograms were analyzed using ModFit LT cell cycle analysis software (Verify Software House, Topsham, ME, USA).

### Apoptosis analysis

Annexin V-FITC Apoptosis Detection Kit I (BD Biosciences) was used to detect apoptotic cells by annexin V staining. Cells were co-incubated with annexin V-fluorescein isothiocynate (FITC) and propidium iodide (PI) and measured by two-color FACS cytometry using BD LSR Fortessa. The percentage of annexin V and PI-positive cells was determined based on the dot plots of FITC vs. PI.

### Western blotting

Attached cells, as well as floating cell in culture medium, were lysed in cell lysis buffer (RIPA buffer (1% NP-40, 50 mM Tris (pH 8.0), 50 mM sodium fluoride, 0.5% sodium deoxycholate, 0.1% SDS, 150 mM NaCI, 2 mM EDTA) with protease inhibitor (Roche) for the liposarcoma cell lines, and kinase buffer (1% NP-40, 50 mM Tris (pH 8.0), 100 mM sodium fluoride, 30 mM sodium pyrophosphate, 2 mM sodium molybdate, 5 mM EDTA, 2 mM sodium vanadate, 10 μg/ml aprotinin, 10 μg/ml leupeptin, and 1 mM phenylmethylsulfonyl fluoride) for the GIST lines). Whole cell lysates were subjected to SDS-PAGE followed by immunoblot with the following antibodies: pKIT Y703, #3073, Cell Signaling Technology Inc., Beverly, MA, USA; pKIT Y721, #3391, Cell Signaling Technology Inc., Beverly, MA, USA; KIT, A4502, DAKO, Carpinteria, CA, USA; pAKT Y308, #9275, Cell Signaling Technology Inc., Beverly, MA, USA; AKT, #9272, Cell Signaling Technology Inc., Beverly, MA, USA; pMAPK(p44/42) Y202/Y204, #9101, Cell Signaling Technology Inc., Beverly, MA, USA; MAPK(p44/42), #4695, Cell Signaling Technology Inc., Beverly, MA, USA; p53, OP43T, Calbiochem, Darmstadt, Germany; MDM2, sc-965, Santa Cruz Biotechnology, Santa Cruz, CA, USA; p21, #2947, Cell Signaling Technology Inc., Beverly, MA, USA; RB, #9309, Cell Signaling Technology Inc.; pRB(Ser807/811), #9308, Cell Signaling Technology Inc.; PARP, #9542, Cell Signaling Technology Inc.; BAX, #2772, Cell Signaling Technology Inc.; α-tubulin, T9026, Sigma-Aldrich, St. Luis, MO, USA; Lamin A/C (JoL3), sc-56140, Santa Cruz Biotechnology, Santa Cruz, CA, USA.

### Gene expression analysis

Total RNA was isolated using the RNeasy Mini Kit (Qiagen, USA) and subsequently reverse-transcribed to synthesize cDNA using High Capacity cDNA Reverse Transcription Kit (Applied Biosystems, Foster City, CA, USA) according to the manufacturer's instruction. Transcript levels were quantified using TaqMan Gene Expression Master Mix (TP53, Hs01034249_m1; CDKN1A (gene encoding p21), Hs00355782_m1; MDM2, Hs01066930_m1; GAPDH, Hs99999905_m1; ACTB, Hs99999903_m1; Applied Biosystems) on a 7900HT Fast Real-Time PCR System and normalized to the average of ACTB and GAPDH. Relative quantification between different samples was determined according to the 2^−ΔΔCt^ [50].

### p53 and RB knockdown by siRNA

Lipofectamine^®^ RNAiMAX (Invitrogen, Carlsbad CA USA) was used for siRNA transfection. Cells were seeded with 2 ml of antibiotic-free medium in a 6-well plate 24 hours prior to the transfection and incubated for another 48 hours after adding 400 μl of RNA-lipid complex for subsequent drug treatment.

### Statistical analysis

Comparisons between groups were made using the two-tailed unpaired *t* test. Differences in mean ± SEM with *P* < 0.05 were considered statistically significant.

## SUPPLEMENTARY MATERIALS FIGURES AND TABLES


