# Supplementary material for: Preclinical activity of selinexor, an inhibitor of XPO1, in sarcoma
Source: Oncotarget. 2016 Feb 24;7(13):16581–92. doi: 10.18632/oncotarget.7667 (PMC4941336; doi:10.18632/oncotarget.7667)
Supplement: Supplementary file 1 [file oncotarget-07-16581-s001.pdf]

Preclinical activity of selinexor, an inhibitor of XPO1, in sarcoma

Supplementary Materials

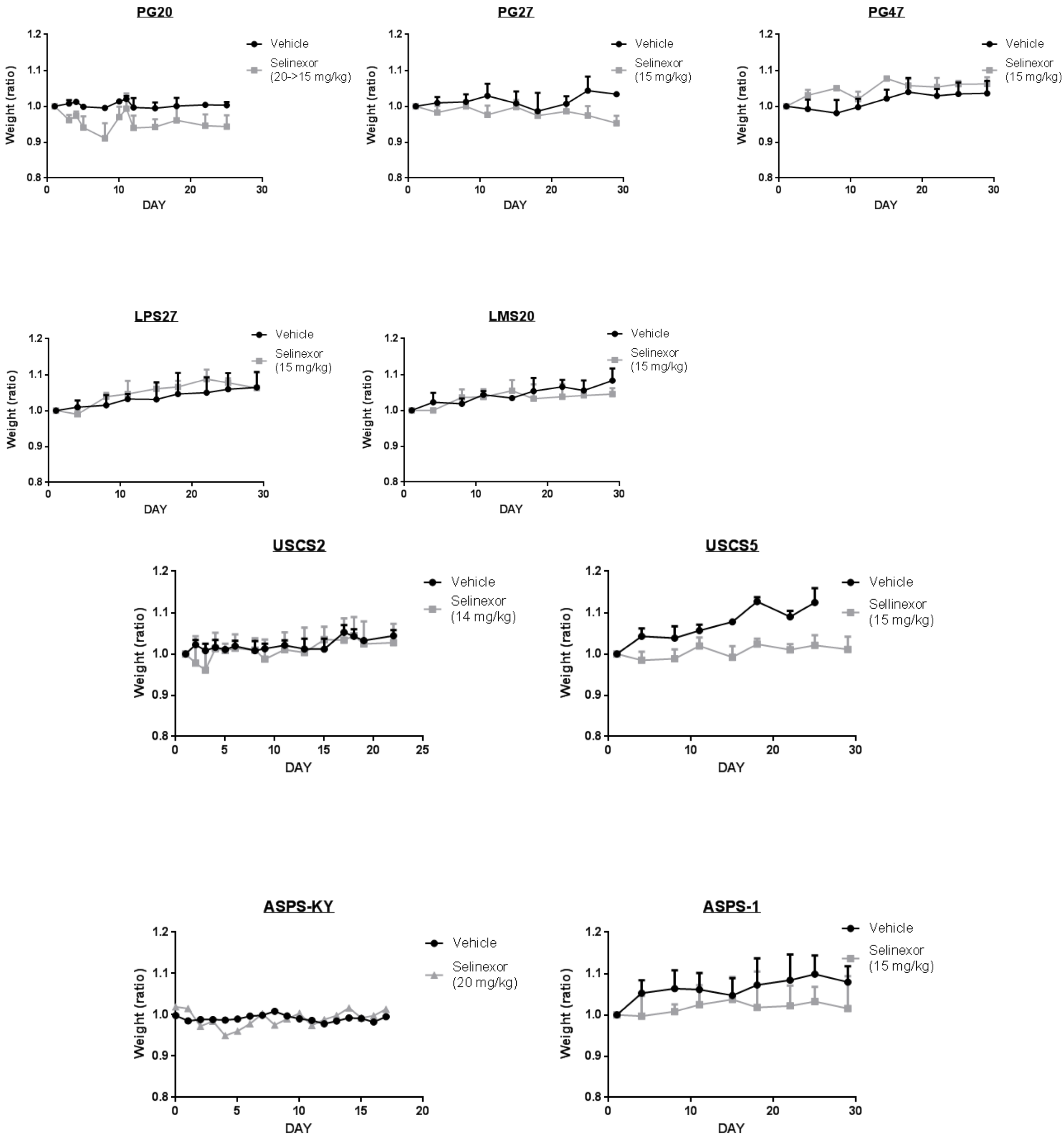

Supplementary Figure S1: Change in mice weight by selinexor treatment in each sarcoma xenograft model. The Y-axis indicates average changes in weight from day 1.

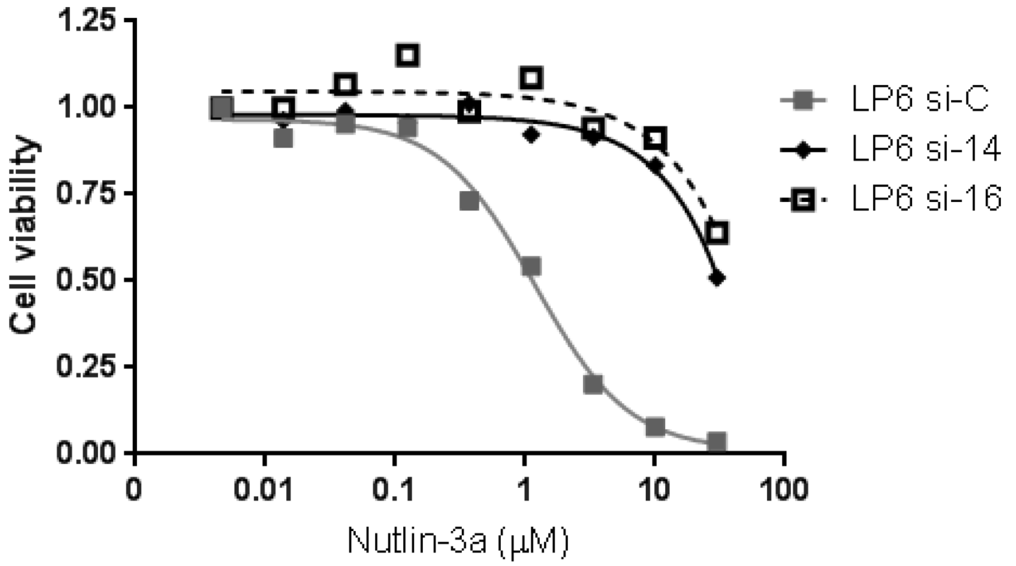

Supplementary Figure S2: Cell viability assay in the p53-knocked down LP6 lines following the 72-hour exposure to the serial concentration of Nutlin-3a.

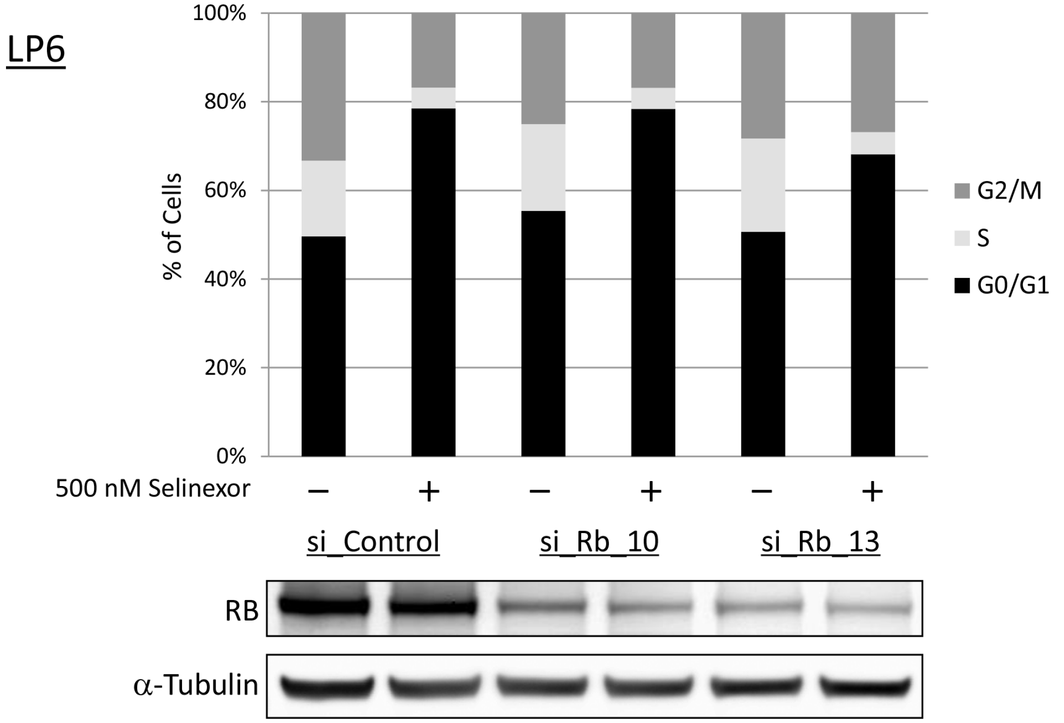

Supplementary Figure S3: Selinexor induced cell cycle arrest in LP6 independently of RB expression. (**A**) Cell cycle analysis by propidium iodide staining in the LP6 line treated with siRNA for RB. The cells were fixed following 24-hour exposure of selinexor. (**B**) Protein expression analysis in the RB-knocked down LP6 lines following 24-hour exposure of selinexor.

Supplementary Table S1: Cell lines for *in vitro* experiments and their sensitivity to selinexor

Histology	Cell line	IC_{50} (nM)	Notes
GIST			
	GIST-T1	69.1	Taguchi et al. Lab Inv 2002 (ref.39)
	GIST-T1/829	91.2	Imanitib-resistant subclone from GIST-T1
	PG27	55.2	
Liposarcoma			
	LP6	51.5	High-copy MDM2 amplification
	LPS12	31.1	Low-copy MDM2 amplification
	LPS141	71.3	High-copy MDM2 amplification
	LPS510	121.6	Mutant p53
Leiomyosarcoma			
	LMS14	43.9	
	LMS17	101.3	
	LMS20	128.4	
Rhabdomyosarcoma			
	RMS559	66.1	
	RMS2	53.3	
Undifferentiated sarcoma			
	USCS2	49.5	
	USCS5	28.8	
	SCPS	218.2	
ASPS			
	ASPS-KY	2099	Hoshino et al. Cancer genetics and cytogenetics 2009 (ref.40)
	ASPS-1	4262	Kenney at al. J Pediatr Hematol Oncol 2011 (ref.41)

*GIST, gastrointestinal stromal tumor; ASPS, alveolar soft part sarcoma.

Supplementary Table S2: Xenograft models

Histology	Name of Model	Type of Model	Notes
GIST			
	PG20	PDX	SDH deficient
	PG27	PDX	Corresponding to the cell line in Table 1
	PG47	PDX	
LPS			
	LPS27	PDX	High-copy MDM2 amplification
Leiomyosarcoma			
	LMS20	PDX	Corresponding to the cell line in Table 1
Undifferentiated sarcoma			
	USCS2	PDX	Corresponding to the cell line in Table 1
	USCS5	PDX	Corresponding to the cell line in Table 1
ASPS			
	ASPS-KY	Cell line Xenografts	Corresponding to the cell line in Supplementary Table 1
	ASPS-1	PDX	Corresponding to the cell line in Supplementary Table 1

*GIST, gastrointestinal stromal tumor; LPS, liposarcoma; ASPS, alveolar soft part sarcoma;
PDX, patient derived xenograft.
